# Mr. Hume on the Eau Medicinale

**Published:** 1811-09

**Authors:** Jos. Hume

**Affiliations:** Long Acre


### *To the Editors of the Medical and Physical Journal.*

GENTLEMEN,

AS it is probable that Mr. Moore's experiments on the *Eau Medicinale D'Husson* may induce a general investigation of this very popular and, perhaps, valuable nostrum, I beg your permission to offer some observations on the subject.

On perusing that gentleman's letter to Dr. Jones, I was struck with a kind of coincidence of opinion, which had led me also to make the same combination, viz. that of *opium* and the root of *white hellebore.* I need make no apology for intruding these remarks, as it will presently appear that I do not arrogate to myself the merit of having discovered the true nature and composition of this celebrated remedy, even should these articles really form the genuine Eau Medicinale.

Soon after the publication of Dr. Jones's pamphlet I was requested by several of my medical friends, particularly by one very respectable Member and Fellow of the London College, to turn my attention to Husson's wine. Having procured what is deemed the true kind, which was then sold in the Haymarket, I immediately began the inquiry, by selecting a number of simples, by way of list, on which I proposed to operate in succession. To detail all the experiments would answer no purpose, for in no one example was I satisfied that we had obtained the end. Here, however, I must confess, my reliance was rather upon smell, taste, colour, and other obvious qualities, than upon the *virtue* of our imitation; and hence the cause of failure, should it ever be fully ascertained that the Eau Medicinale is composed of opium and white hellebore, the *veratrum album.*

Among a number of vegetables on which I had fixed for this synthetic inquiry, the very first, after opium and ipecacuanha, was white hellebore. This was submitted to many trials; it was infused in different wines and in diluted alcohol of various degrees of specific gravity. In all cases, however, the smell and taste seemed to forbid any favorable conclusion, even when combined with opium; and as none of the products had a chance afforded of being internally administered, even in one solitary trial, the farther consideration of this article was totally abandoned.

That we did not persevere so far as Mr. Moore, nor obtain the same fortunate result which has followed that gentleman's labours, must be ascribed chiefly to my own prejudice, in depending too much upon the smell and taste; for the objection, I acknowledge, arose entirely on my side, and not with the worthy individual to whom I allude, and to whom I owe, on many accounts, so much gratitude.

 Now,

Now, had I fully accomplished the object I had in view, a perfect imitation of the Eau Medicinale in efficacy, flavor, taste, and other sensible qualities, and that opium and the veratrum had proved to be the primitive ingredients of which we were in search; I should not, even then, have assumed the title of a discoverer. My reason for declining so much honor would be this, that though these experiments took place above ten months ago, I was then perfectly aware that a composition of opium and white hellebore had been employed in this country, I believe, before Mons. Husson was born; I would, therefore, have acknowledged, as I do now, the source whence I took the hint in favour of the *hellebore* and *opium*, for, I certainly never trusted to the plausible juggle of this nostrum being extracted from one single vegetable only. Here I allude to one of the remedies employed by the famous Ward, the composition of which the public is already in possession of, as all his prescriptions were printed above thirty years ago.

I know that Ward practised in Paris about the year 1736; it is, therefore, probable that he may be the real founder of this nostrum, which, after all, may turn out to be no exotic, but of true English extraction.

Whether Dover's composition of opium with ipecacuanha, or that of Ward's, with white hellebore, claims the priority, I cannot now decide, having no reference at hand on which I can depend; the transition from one to the other is, however, so very palpable, that it greatly diminishes the merit of an imitator or pretender, such as Mons. Husson must be considered. Though Ward is generally reckoned as an empiric, he was certainly not destitute of abilities, for he has left evident proofs of genius behind him. As one example of his acuteness, we may take the modern process for manufacturing sulphuric acid, from nitrate of potash and sulphur, in place of the ancient method, from sulphate of iron only. He employed powerful materials for his medicines, such as antimony, opium, mercury, white hellebore, &c. and consequently the remedies he administered were often successful, though, I am persuaded, seldom if ever dangerous, when his instructions were observed.

I am not prepared to make many observations upon the substitute proposed by Mr. Moore, for the Eau Medicinale D'Husson. I am convinced it deserves a most candid consideration, and that it should be submitted to repeated and varied trials, before it is universally adopted or entirely discarded.

There seems a slight error in the vinum opii employed by Mr. Moore; it is, that of taking *pints* of the wine, in place of *troy pounds*, according to the Edinburgh College. There is indeed a prevailing mistake respecting the old *tinct. thebaica* of of the London Pharmacopœia for 1745; the laudanum of Sydenham; and the vinum opii of the present edition of the London Dispensatory. These are often confounded, though evidently not of the same strength. If the vin. opii be superior to other solutions for the eyes, the spices, I should suppose, ought to be omitted; indeed I can see no advantage whatever in such trifling incumberances as these and many other articles, which frequently enter into officinal preparations, where a mere fractional part of a grain is the full amount in each dose.

In the Edinburgh New Dispensatory, it is observed of the vin. opii, now in use here, that it is the synonime of the tinct. thebaica, according to the Pharm. Lond. 1745. There is assuredly a material difference; the first contains *one* ounce, the last *two* ounces of opium to the pint; besides, to the last, when filtrated, was added one-twentieth part of proof spirit. It is true, in the former the *opium colatum* is ordered, and in our present wine it is *extractum opii*; but here, the danger arises from the variation of *quantity*, for there is little or none in the preparations of the opium itself varying.

White hellebore root was formerly administered as an emetic. We read of *Conradus's vomit* and the *vomiting wine* of *Heurnicus*. The former was prepared with spirit of wine, perhaps *proof* spirit; this was distilled off, and again returned upon the root to complete a tincture. A feather being dipped in this was then washed in a glass of wine, which being swallowed is said to have proved a powerful emetic. The preparation of Heurnicus was merely an infusion of one scruple only of the root in gross powder, in a pint of sack; one spoonful of this acted as an emetic, and half the quantity was occasionally combined with other purgatives to render them more potent.

The occasional examples of violence in the operation, and even of fatal consequences ensuing from the Eau Medicinale, arise, I suspect, from a kind of spontaneous decomposition which takes place in the medicine itself, as it is gradually and constantly depositing a sediment; hence, I would advise, that the phial should always be first shaken, and that it be kept in a cool place and not exposed to the sun. The medicated wines in general are susceptible of this change, and, therefore, the addition of a portion of proof spirit, according to former practice, would be of great use.

If Husson really employs the laudanum of Sydenham, it is likely that he takes the same saffron which we use in England, that is, the *Spanish* saffron, crocus in fœno, as our druggists call it. I believe no respectable practitioner would put *English* saffron into his compounds, for this is sold in cakes, and is avowedly and universally offered at an inferior

 price price, being composed of marygold petals and other such trash. Saffron is, however, of no consequence in the present question, more than what regards the smell and colour of Husson's nostrum.

Of those compounds which resulted from the experiments, that from ipecacuanha and opium seemed to promise most fairly; so much so indeed, that our Dover's powder, occasionally modified by the opium, may very securely be administered as a substitute, by transferring its influence to wine or diluted alcohol. In several cases in which this imitation was administered, the success was equal to any thing ever achieved by the Eau Medicinale; and as ipecacuanha is safe in its action, compared with the white hellebore, there must be frequent opportunities of preferring the former, particularly in extreme debility.

As there is much to commend in Mr. Moore's endeavours to accomplish a complete knowledge of this remedy, especially if he was not aware of Ward's having dealt with white hellebore and opium before Husson's time, I trust the importance of the subject will urge the whole profession to continue the inquiry. The chief reliance, however, is to be placed rather upon its effects as a medicine than its appearance to our senses; I shall therefore leave to those whom it more immediately concerns, to prosecute an inquiry of such vast importance, as an investigation of the *combined* powers of veratrum album and opium upon the human constitution.

JOS. HUME.

*Long Acre, July* 27, 1811.

